# Impact of Facilitation on Cognitive Flow in a Novel Diabetes Management Rehearsal Game for Health Professions Education: Mixed Methods, Open-Label, Superiority Randomized Controlled Trial

**DOI:** 10.2196/54703

**Published:** 2024-07-17

**Authors:** Jun Wen Tan, Gabriel Tan, Xia Lian, Darren Kai Siang Chong, Preman Rajalingam, Rinkoo Dalan, Sreenivasulu Reddy Mogali

**Affiliations:** 1 Lee Kong Chian School of Medicine Nanyang Technological University Singapore Singapore; 2 MOH Holdings Pte Ltd Singapore Singapore; 3 Department of Endocrinology Tan Tock Seng Hospital Singapore Singapore; 4 Institute of Learning Mohammed Bin Rashid University of Medicine and Health Sciences Dubai United Arab Emirates

**Keywords:** serious game, randomized control trial, facilitation, diabetes, diabetes management, flow, education

## Abstract

**Background:**

Though the prevalence of diabetes is set to increase, most serious game solutions typically target patient self-management and education. Few games target health care professions education, and even fewer consider the factors that may increase their efficacies. The impact of facilitation, a prominent feature of health professions education, is examined in the context of a rehearsal-based diabetes management serious game.

**Objective:**

In this mixed methods, open-label, superiority randomized controlled trial, we compare student performance, attitudes, and perceptions of a rehearsal-based diabetes management game for health care professionals.

**Methods:**

Student participants were randomized into 2 groups to play a diabetes management game. The control group played the game alone, and the intervention group played the same game alongside a facilitator tasked to moderate overall challenge levels and address queries. Both groups were administered the Flow Short Scale, a 13-item measure rated on a 7-point Likert scale ranging from 1 (“not at all”) to 7 (“very much”) immediately after the game. Students were then invited to voluntary focus group discussions to elicit their attitudes and perceptions of the game. Findings were subject to between-group comparisons and inductive thematic analysis respectively.

**Results:**

A total of 48 (26 control, 22 intervention) clinical-year undergraduates from the Lee Kong Chian School of Medicine in Singapore participated in this study, with 18 continuing to the focus group discussions. Flow Short Scale results indicated the superiority of the intervention group for overall flow (*t*_46_=–2.17, *P=*.04) and the absorption subdomain (*t*_46_=–2.6, *P=*.01). Qualitative results indicated students viewed facilitation as helpful and appropriate, and were able to identify improvable elements of the game’s theoretical foundations and overall design.

**Conclusions:**

While serious games are efficacious means of rehearsing previously learned knowledge, facilitation allows for their efficiency to be greatly increased. Such increases are likely crucial in the coming years with the increased digitization of health care professions education and the prevalence of diabetes.

**Trial Registration:**

ClinicalTrials.gov NCT05637749; https://www.clinicaltrials.gov/study/NCT05637749

## Introduction

### Background

Diabetes is a chronic disease characterized by sustained high blood glucose and, when left unmanaged, is associated with severe health consequences and premature mortality [1]. Globally, an upward trend in the incidence of the disease has been noted in most regions, driven primarily by type 2 diabetes (T2D) and the prevalence of its associated risk factors [2-4].

This coincides with the increasing complexity of medicine and its need for a skilled workforce capable of taking up new knowledge in constrained time frames [5,6], necessitating the development of new methods to facilitate continuous health care professions education [7].

### Digital Interventions

Despite numerous digital interventions developed in the wake of these needs, notable gaps remain. Educational interventions remain focused on patients with comparatively little for caregivers and health care professionals. Despite favorable outcomes, such interventions may have difficulties sustaining user attention and engagement [8-10]. Reviews of digital diabetes educational interventions suggest their full potential may be stymied by the absence of a human expert to guide the user [11], though it remains unclear if this is a consequence of the rapid uptake of digital technologies and the greater push toward self-reliance.

Jeon and Park [12] noted that self-care apps improve social motivation and behaviors, but not knowledge, behavioral skills, or personal motivation, which instead benefited from in-person interventions, while caregivers actively sought peer-to-peer support to alleviate concerns [13]. In continuous education, learners paradoxically reported great acceptance of distance learning methodologies, while also desiring face-to-face teaching [14,15]. Additional evidence suggests improving motivation and subject attitudes may be more important than enhancing knowledge [16], as observed in interactions with diabetes care services [16]. As a result, despite the student and patient-centered approaches used by modern developments [17-20], evidence of the exact benefits afforded by including a human expert, trainer, or facilitator in these interventions remains insubstantial.

### Serious Games

Serious games may increase patient motivation [21-23], and the management skill of patients and caregivers [12,16,24], in the context of chronic diseases such as diabetes. They are usually defined as games designed for nonrecreational purposes such as education and therapy [25-27], and have enjoyed increasing uptake in both educational and clinical settings due to the ease by which they enhance motivation in their users [25-27]. They are distinct from gamification, which incorporates game-like elements into nongame interventions [28].

Such games are unable to replace qualified professionals, and instead support the promotion of exercise [29,30], deliver diabetes care education [31,32], and facilitate self-management [33,34]. Like their game-free counterparts, they are heavily focused on patients, with few health care professionals or caregivers. The majority appear to be gamified interventions as opposed to serious games, with literature reviews of the past decade returning only a nondigital escape room to improve diabetes management knowledge in pharmacy students and teaching insulin therapy to primary care physicians [35,36].

This dearth likely stems from early attempts to gamify existing methods, which failed due to poor game and instructional design [37,38]. Modern developments are understandably subjected to rigorous validation studies before implementation, and recent reviews of the literature suggest this remains the focus of a vast majority of game-based research—newly developed games are trialed against an established game-free control group, and efficacy is determined by the degree to which the game fulfills its intended purpose [25,39-42]. This user-centric focus, while meritorious, leaves unaddressed the key mechanisms of action responsible for a game’s success, much less to what extent such mechanisms may be controlled to influence how players enjoy or learn from them.

### Roles of Human Educators

Human educators provide emotional intelligence, empathy, and context awareness [43], key drivers of learner engagement and overarching educational outcomes despite advancements in intervention design [44]. With games, prior investigations suggest educators may facilitate learners transiting from passive knowledge retention and learning to encourage self-directed inquiry and active learning [43], as well as provide customized, empathetic, and learner-specific feedback that digital systems may not fully emulate [45].

### Facilitation and Flow

Facilitation is considered by the Promoting Action on Research Implementation in Health Services framework as a process that supports and enables others’ self-improvement and goal attainment [46,47]. In medical education, facilitators are credited for the success of collaborative [48], guided, yet autonomous learning experiences such as team-based learning [49,50]. Success may be attributed to facilitators exhibiting prosocial traits such as empathy, flexibility, authenticity, pragmatism, and credibility [51,52], as well as easing difficulties and keeping students invested in the activity [53-55].

Despite this, facilitation remains a broadly defined concept and the benefits of prosocial traits may not wholly translate to serious game-based interventions. Human experts introduced to games may assume multiple roles such as facilitators, instructors, and mentors [56], among others, with no role being universal due to the myriad roles games may play. Nonetheless, facilitation and moderation are the most likely drivers of success in serious games due to their means of adjusting a game activity to better meet the needs of individual learners.

The meeting of these needs is often a precursor of a flow state, a crucial yet often overlooked feature of serious games. Flow is a cognitive state characterized by absolute attention toward an optimally challenging task and the fluency of one’s actions, seemingly without conscious thought [57-59]. Notably, high rates of flow are associated with a willingness to return and repeat an activity [25,59-61], critical in education where rehearsal facilitates the committing of new knowledge into long-term memory.

Understanding how much influence facilitation may exert on flow generation in serious games is thus key to increasing the efficacy of such games in medical education and further enabling the continuous education of health care professionals in the future.

### Study Aims

To this end, this mixed methods study aims to identify and, where possible, quantify the benefits arising from human-assisted facilitation in digital game-based interventions. This will be accomplished via an open-label superiority randomized controlled trial, then a focus group discussion to elicit greater insight and provide additional context into participant perceptions and attitudes.

A rehearsal-based diabetes management game has been developed for this purpose and includes a special role for a human facilitator tasked with ensuring an optimal game environment for the player.

The quantitative aspect of this study hypothesizes that subjects assigned to a facilitated game group will report statistically significantly higher flow scores than subjects of the unfacilitated group.

## Methods

### Participants

Subjects were recruited from third-, fourth-, and fifth-year medical students undertaking their Bachelor of Medicine and Bachelor of Surgery degree at the Lee Kong Chian School of Medicine, Nanyang Technological University in Singapore, where this study also takes place. These students were selected due to their completion of the endocrinology segments of their internal medicine clinical postings and thus had basic familiarity with diabetes management in both clinical and community health care settings. Recruitment was performed by email advertisements and snowball sampling via word-of-mouth, and the completely voluntary and benefits-free nature of this study was repeatedly stressed. Exclusions included students who had not completed the endocrinology segments of their internal medicine postings, diseases of the eye not including myopia, noticeable psychosocial difficulties, and any other characteristics that may put them at risk while playing the game. All interested participants were instructed to read this study’s information sheet, had the same sheet read to them before consent taking, and were repeatedly informed that they could ask questions and that participation in both the qualitative and quantitative aspects of this study was voluntary.

### Ethical Considerations

Ethical approval was obtained from the Nanyang Technological University Institutional Review Board (IRB-2022-739).

### Theoretical Bases

Before the formal study, informal focus group discussions with clinical-year medical students were conducted to gauge interest, elicit suggestions, and identify key features of the diabetes management game. Following this and subsequent literature reviews [62], it was determined the game intervention would best be developed based on self-determination theory (SDT), flow theory, and experiential learning theory.

SDT posits that an activity becomes intrinsically motivating when the needs of competence, autonomy, and relatedness are met [63]. Competence was addressed by mirroring the behaviors of both nonplayer characters (NPCs) and their ambulatory glucose profiles (AGPs) as closely as possible to case studies students would encounter as part of their education. To address the need for autonomy, players were permitted to manage NPCs in any manner. Relatedness was expected to be established by both the presence of the facilitator and the role the facilitator plays when checking in on the player’s progress.

The facilitator’s role also overlaps with flow theory, as they may adjust game difficulty based on real-time player feedback. Flow refers to a deep cognitive state wherein an individual directs absolute attention toward an optimally challenging task, simultaneously experiencing near-complete control over the activity and a total loss of awareness of the self [57-59]. Such states are associated with increased accomplishment across the breadth of the human developmental life span and, in the context of education and rehearsal, the willingness to return and repeat an activity [25,59-61].

Experiential learning theory stipulates that learning occurs when an individual partakes in the activity or task to be learned as opposed to receiving knowledge through instruction [64], and further overlaps with the aforementioned fulfillment of competence as defined by SDT.

### Game Intervention

The digital diabetes management game is comprised of a single-player management game centered on a 2D community populated with NPCs who all have type 1 diabetes (T1D), T2D, or gestational diabetes (GD). NPCs work, consume meals, and partake in recreational activities within the game environment of their own accord and may not be directly controlled by the player. The player interacts with the game using the mouse and controls the administration of insulin, snacks, and oral medication. Upon clicking the respective buttons, players are presented with a dosage and may adjust it with further mouse clicks before confirming the action. NPCs do not partake in these activities of their own accord.

**Figure 1:**
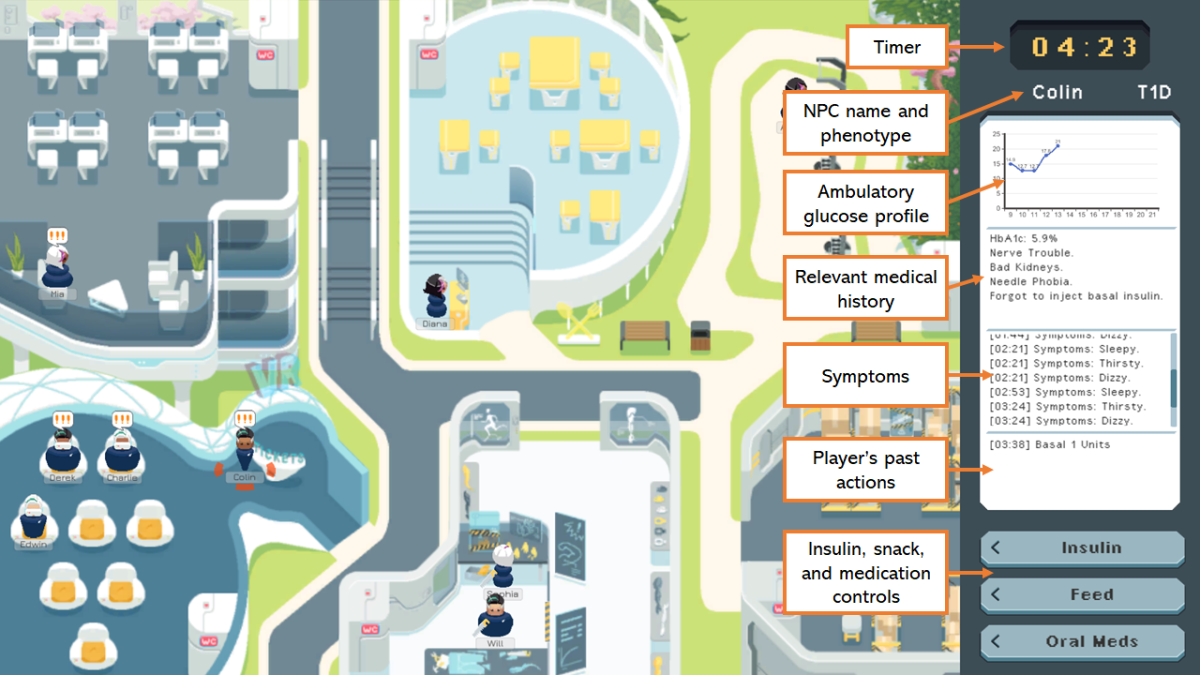
Overview of the game world as viewed by the player, including sidebar display of relevant NPC details. NPC: nonplayer character.

Each NPC possesses individually tracked blood glucose, visible to the player via an AGP, with changes simulated in response to stimuli, such as the physical intensity of current activities, insulin and oral medication dosages, consumption of meals or snacks, and phenotypic characteristics such as insulin resistance. Should extended or severe hyper- or hypoglycemia occur, NPCs will faint, be removed from the game, and the player will be informed that said NPC has been evacuated to an off-site hospital. Upon selecting an NPC, players may view their relevant clinical history, present symptoms, and all past actions they were administered (see Figure 1; a short technical demonstration is included in Multimedia Appendix 1).

Following a tutorial with actual gameplay, the player is given 12 minutes to play the game with 1 real-time minute corresponding to 1 in-game hour. They are tasked to keep the blood glucose levels of all NPCs in an ideal target range (subject to phenotype) as much as possible. With every real-time minute, each NPC’s AGP is updated based on their in-game activities and the player’s inputs to them thus far.

By default, the game begins with 10 NPCs comprising 7 T2D, 2 T1D, and 1 NPC with GD, reflective of the incidence of each phenotype. This number may change based on the actions of a human facilitator, who may access a game in progress from a separate machine. The facilitator is provided the same information as the player and may additionally create new NPCs for the player to manage, remove existing NPCs from play, or as an alternative to removal “freeze” the AGP of existing NPCs such that they need not be managed by the player until “unfrozen” (see Multimedia Appendix 2).

Due to the short duration of gameplay, the role of the facilitator was governed by a strict set of rules that they were not permitted to deviate from during this study. They were not permitted to offer knowledge a player has clearly forgotten (ie, administration of metformin to an NPC with compromised renal function) unless explicitly asked. They were to remind players of the function of game controls if asked, or if the player repeatedly made control-based mistakes (ie, trying to use the right mouse button or keyboard, which have no function). They were to succinctly explain to the player the reason behind an NPC fainting and being conveyed to the hospital should an instance occur (ie, too much bolus insulin 2 hours ago and NPC became hypoglycemic) to avoid disrupting the flow of the game. To minimize the influence of extraneous factors that may result from personal communication styles, the same facilitator was used for all games.

In the first 3 minutes of a facilitated game, the player will play the game solo with the facilitator remaining out of sight and taking no actions. On the third minute, and every 2 minutes after, the facilitator will approach the player and ask, “How are you faring?” The player would then indicate how well they are handling the present difficulty and if they desire a change in the number of active NPCs.

### Study Design

This study used the CONSORT (Consolidated Standards of Reporting Trials) as guidelines and was conceptualized as an open-label, superiority randomized controlled trial (Multimedia Appendix 3). Following consent by the on-site member of this study’s team, subjects were briefed on their objectives, given the tutorial, and allowed to familiarize themselves with playing the game until they had no further questions. Subjects were then randomized into either the facilitated intervention or facilitator-free control via simple randomization using Sealed Envelope, a secure web-based randomization service based in London, the United Kingdom [65], that allowed for allocations to be concealed from all parties until after a subject was enrolled and ready to partake in the intervention. Aside from the secure password to enable each randomization, subjects were permitted to view the result of their randomization.

Should the subject be randomized into the facilitated intervention group, the role of the facilitator would be repeated to them, and any last-minute questions answered. Otherwise, the facilitator would ensure the start of the game and then exit this study’s site until the control’s game had elapsed. The game itself was app-based and played on an internet-enabled university laptop belonging to this study’s team, with the facilitator remotely joining from a separate laptop on the same network.

**Figure 2:**
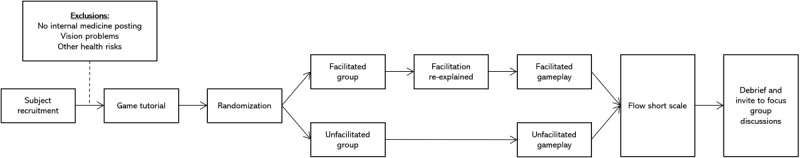
Overview of the study design.

Upon conclusion of the gameplay, subjects were immediately administered the Flow Short Scale (FSS), issued an e-voucher as an inconvenience fee, and invited to the focus group discussion (see Figure 2). Subjects who attended the focus group discussion were reminded that the focus group discussions would be recorded for transcription by a third-party transcription company and any subject unwilling to consent again was allowed to leave. Subjects were then shown and allowed to refamiliarize themselves with the game through play. The guiding questions of the focus group comprised: (1) Do you recall becoming really immersed in the game? What were you doing just before? (2) What did the game do to capture and retain your attention for extended time periods? (3) If you could improve the game to make it clearer and more balanced, what would you do?

### Flow Short Scale

Following gameplay, subjects of both groups were administered the FSS. The FSS consists of 13 items on a 7-point Likert scale ranging from 1 (“not at all”) to 7 (“very much”) [66]. The FSS demonstrates good construct validity, psychometric properties, and a stable 3-factor structure comprising fluency of performance, absorption by activity, and perceived importance or outcome importance of said activity [67]. Flow itself comprises the first 10 items and the domains of fluency and absorption [66]. The scale is typically administered immediately after an activity as a retrospective measure of flow in said activity, and was, for this study, hosted on a university-secured Google Forms and transmitted to subjects via a QR code.

### Power

Power calculations were performed via Sealed Envelope sample size calculations [68], with an α level of 5%, 90% power, and the anticipated control group means of 4 (0.5 above the mean of 3.5 due to games innately being conduits of flow) and anticipated intervention means of 5.05 (15% higher than the control), and an SD of 1. The increase of 15% was based on the results of prior studies comparing game-based interventions and established nongame controls on the results of the FSS [69,70]. An estimated 22 subjects per group for an overall 44 was expected.

### Data Analysis

Quantitative analysis will comprise group comparisons of either discrete or continuous data drawn from FSS and in-game scores. If normally distributed, data will be analyzed via independent samples *t* tests (2-tailed across the board), with Welch correction performed should equality of variances not be observed. Data that are not normally distributed will be compared with Mann-Whitney *U* tests instead. Effect sizes will be calculated for FSS data to better visualize the degree of impact facilitation has on flow generation. Exploratory examinations of all data collected automatically by the diabetes management game will be performed to identify any notable differences between groups. The threshold for statistical significance is set for <.05 per convention, and will be performed using R (version 4.3.1; R Foundation for Statistical Computing).

Due to the exploratory aspect of this study and the novelty of the proposed intervention design, transcribed focus group discussions were subject to inductive thematic analysis per the guidelines of Braun and Clarke [71,72]. A reflexive approach was adopted [72], with transcriptions performed by 2 authors (JWT and DKSC) with a third (SRM) acting as a referee. Of the 3, JWT and DKSC have prior histories of playing video games; only JWT plays recreational video games regularly, DKSC no longer plays video games, while SRM has a very limited history of playing video games. To minimize transference of bias, no communication or input was permitted during the initial stages of analyses. Both JWT and DKSC would first read the transcripts until familiarized, then begin a preliminary coding process and generate a series of initial themes. At this stage, prospective codes were deemed to be anything that appeared to be related to student perceptions of advantages afforded by facilitation, disadvantages resulting from its absence, and any other factor not accounted for by the research question but deemed serendipitous by the coders. Upon completion, these initial themes were then individually reviewed against previously identified codes and refined as necessary. Each analysis was then compared and discussed, with all differences highlighted for discussion and resolution with the referee. Themes were then cross-checked to ensure they represented clear patterns, and iteratively reviewed until each possessed a distinct scope with minimal overlap.

## Results

### Participants

**Figure 3:**
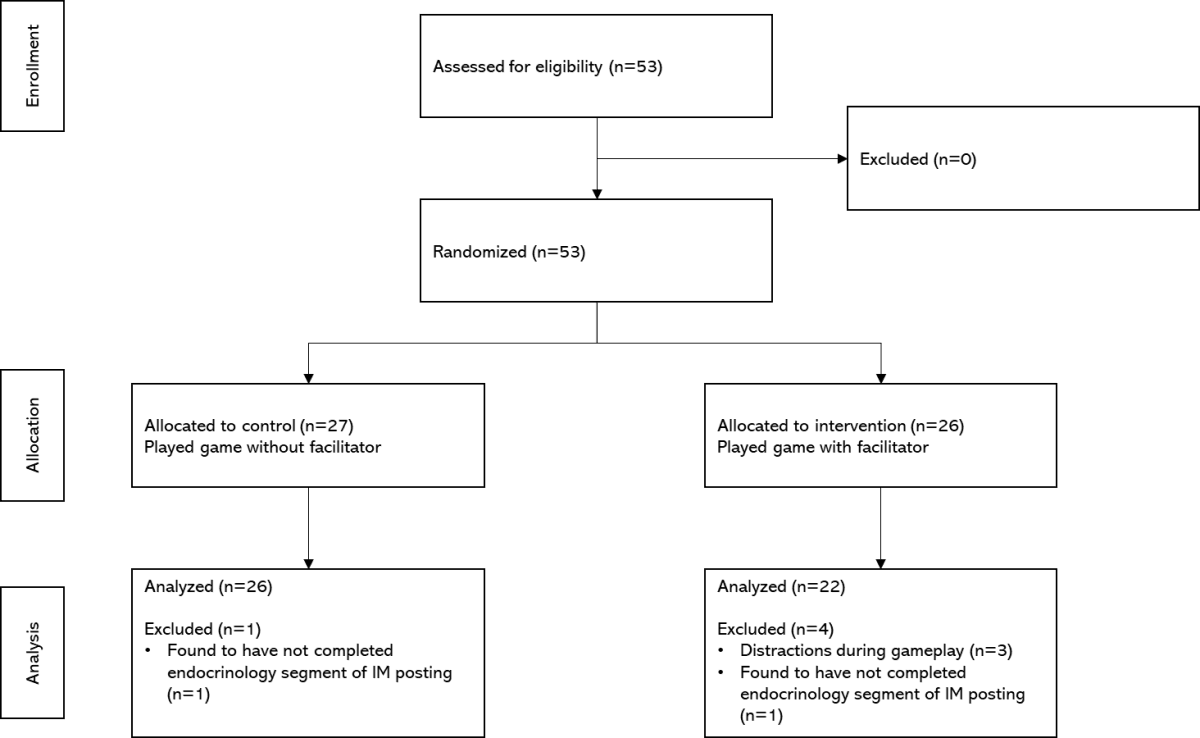
CONSORT flow diagram for participant allocation. CONSORT: Consolidated Standards of Reporting Trials; IM: internal medicine.

Of the 53 subjects recruited into this study, 5 were eventually excluded due to being visibly distracted (ie, mobile phones) while playing the game or were found to have not actually completed the endocrinology segments of their internal medicine postings. A total of 48 subjects were thus included; comprising 26 control and 22 intervention randomizations (see Figure 3 for CONSORT flowchart).

Of the 48 analyzed subjects, there were 25 (52.1%) male and 23 (47.9%) female students aged between 21 and 25 (mean 22.44, SD 1.17) years. Control group subjects comprised 13 male and female students, while intervention group subjects comprised 12 male and 10 female students. The mean ages for both groups were 22.5 (SD 1.36) and 22.4 (SD 0.91) years respectively.

A total of 18 students continued on to the focus group discussions. Of these, 5 male and 5 female students were from the control group, and 5 male and 3 female students were from the intervention. Three sessions were conducted comprising 4 subjects (3 male, 1 female) with 1 control and 3 intervention assignments, 8 subjects (4 male and 4 female) with 6 control and 2 intervention assignments, and 6 subjects (3 male and 3 female), with 3 control and 3 intervention assignments. Table 1 presents an overview of participant demographics for each group in this study.

**Table 1:** Overview of participant demographics for both the randomized controlled trial and the focus group discussions.

Characteristics			Control		Intervention
**Game randomized controlled trial**					
	Total participants (n)	26		22	
	Male, n (%)	13 (50)		12 (54.5)	
	Female, n (%)	13 (50)		10 (45.5)	
	Age (years), mean (SD)	22.5 (1.36)		22.4 (0.91)	
**Focus group discussions**					
	Total participants (n)	10		8	
	Male, n (%)	5 (50)		5 (62.5)	
	Female, n (%)	5 (50)		3 (37.5)	
	Age (years), mean (SD)	21.7 (0.48)		22 (0.76)	

### Between-Group Comparisons

Table 2 presents a summary of the performance of between-group comparisons. Normal distribution of data and homogeneity of variances were observed. Independent samples *t* tests were performed across FSS data, and results indicated superiority for overall flow (*t*_46_=–2.17, *P=*.04), weighted primarily on the absorption subdomain (*t*_46_=–2.6, *P=*.01) of the intervention group. No significant differences were observed between the fluency subdomain F and importance (*t*_46_=–0.2, *P=*.84). These results suggest a moderate to high degree of flow for both the intervention and control groups, with notably high absorption for the intervention group, and are supported by the moderate to relatively high effect sizes of 0.63 for overall flow and 0.75 for absorption respectively.

**Table 2:** Summary of *t* tests and effect size calculations between the control (unfacilitated) and intervention (facilitated) conditions.

Variables	Control, mean (SD)	Intervention, mean (SD)	*t* test^a^ (*df*)	*P* value	Effect size, Cohen *d*
Overall flow	4.4 (0.89)	4.95 (0.85)	–2.17 (46)	.04	0.63
Fluency	4.03 (1.16)	4.52 (1.06)	–1.5 (46)	.14	0.44
Absorption	4.96 (0.83)	5.6 (0.87)	–2.6 (46)	.01	0.75
Importance	4.26 (1.39)	4.35 (1.78)	–0.2 (46)	.84	0.06
Mean hours of ideal glucose	2.66 (1.36)	2.35 (1.09)	0.86 (46)	.4	N/A^b^
Metformin errors	2.77 (1.88)	2.73 (1.67)	0.08 (46)	.94	N/A
Number of evacuations	1.58 (1.94)	0.64 (0.95)	2.07 (46)	.04	N/A

^a^Two-tailed.

^b^N/A: Not applicable.

Gameplay analytics indicated no significant differences between hours NPCs spent at an ideal blood glucose level (*t*_46_=–0.86, *P=*.4) and the number of NPCs administered metformin when contraindicated (*t*_46_=0.08, *P=*.94). Ideal blood glucose was defined as 5 to 9 mmol/L for T1D and T2D, and 5 to 7 mmol/L for GD.

A significant difference was noted for the number of NPCs requiring medical evacuation, which occurs when they have extended hyper- or hypoglycemia and faint, in favor of the intervention group (*t*_46_=2.07, *P=*.04).

### Thematic Analysis

The focus group discussions elicited a broad range of student insights and perceptions, evidence in support of the theoretical foundations that afford such games their efficacy, perceived advantages of human-directed moderation of game difficulty, and suggestions on how to improve the intervention. Focus group discussion transcripts were anonymized to preserve the confidentiality of the research data and all mention of names were removed. Although the sessions were conducted in English, all participants were Singaporean and frequently communicated in Singlish—an English-based creole that, while comprising almost entirely of English words, uses a grammatical structure that deviates heavily from standard English. Sessions were transcribed verbatim to avoid accidental changes in meaning and retained Singlish terms such as “ya,” usually an analogue of “yes” but sometimes occurs as general affirmation, and the ubiquitous particle “lah,” typically found at the end of sentences that, when spoken with an appropriate tone, may modify an utterance akin to the use of adverbs in standard English.

### Perceptions on Facilitation

Thematic analysis of the focus group discussions indicated facilitation was mostly helpful, and that students felt a sensation of safety and were more likely to undertake greater challenges as a result.

Yeah, I think it was quite… It was sort of like a safety blanket, you know?

Student 1A

I think I just felt like if anything happened I can go to the facilitator, like, hi, can you help? Can you take out one person? That would be like the guy over there.

Yeah, same. [facilitator’s name] actually was basically my lifeline when I think about it.

Student 1B

Conversely, unfacilitated students experienced increased challenge and performance went down when this was too much for their skill levels, and desired facilitation when this occurred.

Okay, so it was challenging, but it was very frustrating, and I didn’t know what I could do to resolve it.

Student 1C

Yeah. I think a facilitator would have been good or at least there would be, like, instructions on the screen, lah.

Student 1C

### Perceptions of Support for SDT

Support for SDT was deemed as features already present or features that if added would support the theory in the context of meeting the needs of competence, autonomy, and relatedness. Students who perceived themselves struggling with underperformance, actual or otherwise, requested additional modifications to the game beyond what the facilitator was capable of.

But yeah, it will be better if there’s, like, a tutorial or something from the easy levels to high levels, like that, yeah.

Student 1C

And then, after that, it’s like, you’re frustrated ’cause, like, your course is not really going well.

Student 1B

Feelings of autonomy were noted to already be present due to the numerous means of resolving problems and that actions were free of true consequences.

But because I didn’t feel like there was any serious consequence, because it was a game, so I thought it was quite fun.

Student 2D

Perceptions of relatedness were most prevalent during attempts to involve peers as fellow participants and included comparisons to popular cooperative recreational games.

So, instead of it being confined to just the cafeteria and the outdoor exercise area, we could have the opportunity to explore more places…

I’m thinking like an Overcooked kind of thing, like, different islands.

Students 1A and 1B

### Perceptions of Support for Flow Theory

Discussions of the game activity suggested students who were facilitated were more likely to experience an altered perception of time despite there being a clock in the game.

I think for me, I didn’t really care too much about the time. So, like, when [facilitator’s name] stopped me, I eh 12 minutes already?

Student 3B

But it was a fun experience. I felt engaged, because every minute I would check everyone’s [blood glucose]. So, I did not realise, like, that time had passed.

Student 2D

When queried, students were retrospectively aware of becoming completely absorbed in the activity to the point of forgetting about the facilitator’s presence, despite the regular check-ins.

It’s like I don’t have the mental capacity to focus on anything else.

Student 3C

I think I completely forgot that I can ask the facilitator questions.

Student 3A

I just kept clicking around each patient to see where it was going, and the threads, and whatnot. I think that that’s what really kept my attention most of the time…[Student 2C]

### Perceptions of Game Design Elements

Students generally perceived the game as fun, enjoyable, and an appropriate means of revising diabetes management knowledge. The intervention was perceived as both challenging and a safe space in which to commit mistakes harmlessly.

Especially fun cause there’s the whole threat of them possibly dying in the hospital makes it, like, more exciting and more fun to play.

Student 2C

But because I didn’t feel like there was any serious consequence, because it was a game, so I thought it was quite fun.

Student 2D

Student discussions frequently resulted in feedback and disagreements on the merits of said feedback were likely evidence of the specific needs of students playing the game.

I think they should stop moving. Like, moving doesn’t help anything and it doesn’t add anything.

I like the moving though.

The moving was fun lah you just keep chasing the guy around.

Students 1B, 1A, and 1D

Despite not being prompted, students were able to raise requests for changes to better align the game with SDT and flow theory. Changes in line with SDT from the game design perspective primarily focused on being able to play the game with other people and meet the need for relatedness.

Like, you can play with a friend… Unless, I don’t know, there’s some multi-player function introduced.

Student 1A

Changes in line with flow theory focused on how the game should have better-presented information to students, ranging from succinct to full and detailed explanations.

Maybe at the start, before you start playing, that there’s a screen that shows everybody with all their conditions.

Student 3E

So, either working on a different way of showing they were thirsty, like maybe an icon that shows that they’re thirsty instead…

Student 2G

### Additional Findings

Although not the focus of this study, it was noted that certain student characteristics may exert some influence over the degree they engage with the game activity and facilitator. Further, 1 student indicated altruistic motives as a driver of engagement.

I was pretty immersed in the game, and especially with the fact when the people started dying and getting hospitalised. I think, like, when… Once that’s happening, then, yeah, like, oh no, and then you feel more immersed in the game, because you want to keep everyone else alive.

Student 3E

Students who appeared more forgetful than their peers were also likely to express frustration that inhibits engagement.

…apparently the endocrinology emphasised that during multiple tutorials, but I don’t have any recollection of that at all.

Student 3B

## Discussion

### Principal Findings

Results from the between-comparisons indicate support for the hypothesis; the facilitated group is superior, based on the moderate to fairly large effect sizes, to the control in terms of overall flow and the subdomain of absorption, but not fluency. Analyses of the focus group discussions suggest that, beyond flow, ideal conditions for flow were supported by the perception of safety and its related willingness to push oneself toward greater challenge. These findings were unlikely results of differing competencies between groups, evidenced by the nonsignificant differences in time NPCs spent at healthy blood glucose and the number of inappropriate administrations of metformin. Additionally, students of the intervention group almost universally forgot they were allowed to clarify the effects of medication and refrained from doing so as a result. This forgetting to ask for help renders the lower rate of medical evacuation to be most likely the result of the intervention group’s difficulty adjustment as opposed to the facilitator reminding students of the effects of medication. This is likely the result of intense concentration on the game activity resulting from a high flow state as indicated by student reports of total attention being given to the activity. The higher flow scores of the intervention group also offer support to the notion that facilitation confers tangible benefits that result in increased engagement [56,73]. Due to substantial correlations between flow and intrinsic motivation [74], it is likely that students would be more willing to engage in a facilitated serious game due to interest and its enjoyability, and thus be more likely to re-engage in the activity without the need for an incentive [63].

Analyses of the focus group discussions have also indicated substantial support for flow theory and SDT as theoretical foundations of serious games in this design. Even when unprompted, students frequently requested the modification of game features that would circumvent a specific difficulty they experienced, only for other students to disagree with the merits of said requests. This both highlights the facilitator’s role in helping students circumvent specific difficulties, and flow theory’s need for a balance between the challenge of the activity and the learner’s perceived skill [58]. Similarly, reports of feeling safe when paired with a facilitator likely stem from the need for relatedness as defined by SDT. Due to students’ unfamiliarity with the facilitator, this is likely in the context of a mentor-mentee relationship as opposed to friendships [73,75] and is likely not observable in serious games featuring dynamic game balancing as the sole option for difficulty modulation [76]. While it could not be readily determined from the focus group discussions, there is a possibility that perceptions of safety stemmed from fulfilling the need for autonomy, due to a facilitated game affording students a means of exercising greater control over their learning environment [56,77].

While the use of games in medical education and training is not new, an embedded role for a facilitator remains uncommon even in nonmedical literature, with self-selected or dynamic game balancing remaining the common form of difficulty modulation [76]. As a result, the design of the present intervention appears unique to the best of the authors’ knowledge in examinations of both gray and peer-reviewed literature. The inclusion of a facilitator remains beneficial particularly to beginners and players exhibiting low confidence, as evidenced by student perceptions of a “safety blanket.” Comparable studies include a diabetes education and self-management study that paired young patients with mentors to alleviate the emotional stresses of adolescence and reduced the socioeconomic costs of the disease [75], a game-based learning tool for children with content that could easily be modified by an educator resulted in greatly increased student engagement and willingness to participate [78], and a qualitative study that suggested the facilitators required a mix of managerial and technical skills to blend away difficulties faced by students such that they may fully engage in a game-based activity [56]. This study’s design nonetheless aligns with facilitation’s role to support, give, and encourage learners as opposed to teaching the content in question, itself key to simulation and game-based interventions to which the diabetes management game belongs [79]. In addition, the results suggest some relation to the sociocultural theory of cognitive development as defined by Vygotsky, which posits that learning is a social process occurring primarily through interactions between a learner and an expert mentor [80]. Though the theory was not central to the development of the intervention, it may imply that learners used to learning with facilitators may be more receptive to the intervention than those used to learning on their own.

Serious games for diabetes management are almost universally directed at those affected by the disease and understandably target behavioral change as the ultimate goal of education [81]. This study presents one of the few serious games for use in health care education that includes an option for facilitation, itself understudied and uncommon to games even beyond the health care setting [56,79], but is limited by a lack of suitable comparators in the medical literature. In engineering education, it was noted that no 1-model decided the best means of facilitating a serious game, but that learning tended toward being experiential in nature [56], limiting its comparable applicability with the content of the diabetes management game, which falls primarily between the “Knows” and “Knows How” tiers of Miller’s pyramid [82,83]. However, due to the clear distinction between the role of the facilitator and the game intervention itself, it should be possible for the facilitator’s role to be generalized to other topics within health professions education. This may be further supported by the game’s focus on the lower tiers of Miller’s pyramid, and that the standalone game may be played relatively effectively without facilitation.

Finally, though the intervention was intended as a supplementary rehearsal tool, it appears to be a suitable means of formative assessment when played without facilitation [84], and for the rapid detection of learning gaps and their prevalence in a cohort.

### Limitations

This study’s inclusion of a dedicated role for a facilitator in a rehearsal-focused game-based intervention appears to be unique. This role, and its ability to moderate a learner’s gameplay in real-time, does not feature in the recent literature and limits comparisons. This study’s focus on ascertaining the benefits of facilitation in the context of a rehearsal-based game for diabetes management knowledge is itself a limitation, for the exact long-term effects on the topic cannot be determined without a follow-up long-term study involving a version of the game refined after player feedback. Though sufficiently powered and based on a strong theoretical foundation, this study nonetheless presents a sample drawn from a single institution and cohort of students.

Similarly, triangulating the findings via studies involving the same methods of facilitation, but with different topics within health professions education, will help determine the long-term effects of facilitation and its benefits to medicine. The semiexploratory nature of the intervention, and the use of a one-to-one facilitator-student ratio means the intervention cannot be sustainably upscaled without first determining an optional means of increasing the ratio, limiting its deployment to smaller scales. Finally, an element of self-selection bias favoring students with preexisting interests in video games may have been present due to the voluntary nature of this study.

### Conclusions

The inclusion of a facilitator in a rehearsal-based medical serious game can increase the degree to which a student may engage in an activity and elicit sensations of safety with the corresponding willingness to embrace greater challenge. The benefits appear particularly notable for participants who are beginners or unconfident in their abilities and are likely to be the result of facilitators easing difficulties to greater align the participant with the conditions of flow and SDT.

## Appendix

Multimedia Appendix 1 Technical demonstration of the diabetes management game’s control system. All participants will interact with the game in this manner.

Multimedia Appendix 2 A sample video demonstrating the freezing, unfreezing, removal, and addition of a non-player character (NPC) by a facilitator. Participants assigned to the facilitated gameplay will experience these.

Multimedia Appendix 3 CONSORT-eHEALTH checklist (V 1.6.1).

## Abbreviations

AGP: ambulatory glucose profile
CONSORT: Consolidated Standards of Reporting Trials
FSS: Flow Short Scale
GD: gestational diabetes
NPC: nonplayer character
SDT: self-determination theory
T1D: type 1 diabetes
T2D: type 2 diabetes

## Data Availability

The data sets generated and analyzed during this study are available from the corresponding author upon reasonable request.
